# Assessing Stigma, Disclosure Regret and Posttraumatic Growth in People Living with HIV

**DOI:** 10.1007/s10461-018-2230-2

**Published:** 2018-07-20

**Authors:** Bridget Dibb

**Affiliations:** School of Psychology, University of Surrey, Guildford, Surrey GU2 7XH UK

**Keywords:** Adjustment, Disclosure regret, HIV, Perceived stigma, Posttraumatic growth

## Abstract

Posttraumatic growth after a diagnosis of HIV is positively associated with adjustment, yet stigma and disclosure regret are negatively associated with adjustment. Research into whether posttraumatic growth is experienced while perceiving stigma and disclosure regret is still growing. This study aimed to determine whether posttraumatic growth maintains a positive relation with life satisfaction after controlling for disclosure regret and perceived stigma. Using a cross-sectional design, a questionnaire measuring life satisfaction, health status, depression, posttraumatic growth, disease severity, perceived stigma, disclosure regret, and demographical information was completed by 73 people living with HIV (PLWH). Results showed that all participants had disclosed to at least one person. Regression results showed that after controlling for other variables, including stigma and disclosure regret, posttraumatic growth was positively associated with life satisfaction. The importance of the relation of posttraumatic growth with subjective measures of adjustment may be important for interventions aimed at supporting PLWH.

## Resumen

El crecimiento postraumático después de un diagnóstico de VIH está asociado positivamente con el ajuste; sin embargo, el estigma y el arrepentimiento por la revelación se asocian negativamente con el ajuste. La investigación sobre si se experimenta crecimiento positivo cuando se percibe estigma y arrepentimiento va en aumento. El objetivo de este estudio fue determinar si el crecimiento positivo mantiene una relación positiva con la satisfacción de vida cuando se controlan el arrepentimiento por la revelación y el estigma percibido. Utilizando un diseño transversal, 73 personas con VIH completaron un cuestionario que medía la satisfacción de vida, el estado de salud, la depresión, el crecimiento postraumático, la gravedad de la enfermedad, el estigma percibido, el arrepentimiento por la revelación y datos demográficos. Los resultados mostraron que todos los participantes se habían “abierto” al menos a una persona. Los resultados de la regresión mostraron que, después de controlar otras variables tales como el estigma y el arrepentimiento por la revelación, el crecimiento postraumático se asociaba positivamente con la satisfacción de vida. La importancia de la relación del crecimiento postraumático con medidas subjetivas de ajuste puede ser importante para las intervenciones destinadas a apoyar a personas con VIH.

## Introduction

Receiving an HIV diagnosis, usually a stressful event, can be complicated by the disclosure of one’s status to others. As disclosure exposes the individual to potential stigma and stigma has been found to have a negative influence on quality of life, disclosure regret may be experienced. Posttraumatic growth has also been found to be experienced by PLWH and research shows this has a positive effect on health outcomes. However, there is a paucity of research looking at posttraumatic growth, perceived stigma and disclosure regret together, meaning that a full understanding of the relationship is not clear [1, 2]. This study sought to determine whether posttraumatic growth continues to have a positive effect on adjustment once perceived stigma and disclosure regret are accounted for.

HIV is a stigmatising condition and experiences include embarrassment, rejection by others, feelings of isolation, and depression [3]. Disclosure of the diagnosis to others has also been described as stressful [3, 4] and has often resulted in a fear of stigma in anticipation of potential future stigma [4–6]. Disclosure may lead to feelings of regret about disclosing as negative effects include high levels of distress, low levels of self-esteem and social support [4, 7], increased depression [8, 9], and poorer mental and physical health [10]. A fear of stigma may also lead to non-disclosure of diagnosis [7, 11]. The decision of whether to disclose or not has been described as a dilemma [11] where the individual weighs up the risk of experiencing stigma (discrimination and feelings of shame) against the potential of receiving positive support. Tackling the reluctance to disclose one’s status [5] is key as there is evidence that non-disclosure is linked to poorer health [12] and disclosing has been found to be associated with positive aspects for health, such as, a better perception of physical health, lower depression scores, and better mental health [13–15].

Growing evidence shows that the experience of a traumatic event, although accompanied by many negative consequences, is also often associated with perceptions of positive change or growth. This positive growth (also referred to as posttraumatic growth and benefit finding) has been found to occur after traumatic events such as traumatic brain injury [16] and exposure to traumatic wartime events [17]. Moreover, receiving a diagnosis of a chronic illness can also serve as a catalyst for perceiving posttraumatic growth, for example, cancer [18–21], Ménière’s disease [22, 23], myocardial infarction [21], and also HIV [24–26]. However, little is understood about what posttraumatic growth is perceived when negative experiences such as stigma and disclosure regret are also experienced.

Posttraumatic growth is associated with better adjustment, for example, PLWH who perceive more posttraumatic growth have shown better adjustment, satisfaction with life and quality of life [27, 28], and lower depression [25]. Stress-buffering effects have also been shown, countering the depression and anxiety associated with HIV [29]. Qualitative evidence has shown that posttraumatic growth in PLWH occurs in terms of re-evaluating life and becoming more religious [5], perceiving changes in relationships [26], growth in spirituality [26, 30], positively interpreting their situation [24], and changes in the value of life, goals, and health behaviours [24, 26].

Two theories have linked posttraumatic growth to adjustment where growth is perceived in response to the crisis and is a way of coping with and adjusting to the crisis. In this way posttraumatic growth can be both an outcome in response to a trauma and a mechanism of the adjustment process itself [31]. Taylor’s [32] Cognitive Adaptation Theory (CAT) proposes that after a trauma a person experiences “a search for meaning”, which includes perceiving posttraumatic growth where individuals perceive something positive as a result of their trauma, which allows them to give meaning to their situation. The Organismic Valuing Theory (OVT) [33] offers an explanation for how the growth may occur. It proposes that a stressful event shatters our understanding of our self-identity and the subsequent restructuring process involves growth in psychological well-being through assimilation and accommodation of information. This results in the development of a new self-identity; a process which works best in a supportive social environment. However, the experience of stigma means that the social environment may not always be positive and the studies discussed above did not measure perceived stigma or disclosure regret, meaning that the association between posttraumatic growth, disclosure regret and perceived stigma is as yet unknown.

Knowledge of the effects of posttraumatic growth is expanding, however, the relationship between perceived stigma, disclosure regret and posttraumatic growth is still developing. This research has shown a negative association of perceived stigma with posttraumatic growth [1, 2, 34]. Some studies also looked at the relation of disclosure and posttraumatic growth and found a positive relation, however, disclosure regret was not measured [2]. As posttraumatic growth is proposed to occur best within a supportive environment, growth in stigmatizing conditions and where regret is experienced may be impeded. Knowledge of the relation of posttraumatic growth, stigma and disclosure regret may benefit PLWH who have not yet disclosed and those who support and care for this group. The aim of this study was to address the question of whether posttraumatic growth is associated with adjustment after controlling for the negative effects of perceived stigma and disclosure regret.

## Method

### Design

A cross-sectional survey design was used to explore whether posttraumatic growth has a positive relation with adjustment, after controlling for perceived stigma and disclosure regret.

### Participants and Procedure

After receiving ethical approval from the University Psychology Research Ethics Committee the questionnaire was made available on the internet via Survey Monkey. Participants were recruited through HIV support group social network pages where a link to the electronic questionnaire was posted and participants could click directly on the link to access the electronic questionnaire. Participants were not compensated for their participation. Participants were eligible if they were over 18 years of age, could understand English, and had received a diagnosis of HIV. Seventy-three participants completed the questionnaires and the median completion time was 23 min. Sixty-four percent (n = 44) considered themselves to be white or Caucasian, 21.7% (n = 15) considered themselves to be Black African or Caribbean, 8.7% (n = 6) considered themselves to be Asian, and 5.8% (n = 4) considered themselves to be of mixed heritage.

### Measures

Adjustment was measured with a measure of satisfaction with life. Other measures included factors known to influence adjustment (demographical information, physical symptoms, mental and physical functioning and depression) and the predictor variables (posttraumatic growth and stigma (perceived stigma and disclosure regret)).

#### Demographical Information

Demographical Information measures included age, gender (M = 1; F = 2), marital status, sexuality, ethnicity, time since diagnosis, and time since disclosure. Disclosure was measured by the number of people disclosed to (1, < 5, < 10, < 15, < 20, < 30, and > 50), and everybody had disclosed to at least one person. Two open-ended questions (with a free-text response format) asked the participant for the reason they had disclosed their status and who they first disclosed to.

#### Life Satisfaction

Life satisfaction was measured with the Life Satisfaction Scale [35] which consists of 5 items answered on a 7-point Likert scale (1- strongly disagree – 7- strongly agree) where a high score indicates more satisfaction with life. A total score is calculated and the alpha for this scale in this study was 0.83.

#### Disease Severity

Disease severity was measured using Holzemer’s 26-item HIV symptom scale for research [36] which measures both the number and severity of symptoms on a four point scale (none, mild, moderate, and severe). A high score indicates more symptoms and the alpha score in this study in this study was 0.96.

#### Mental and Physical Functioning

The MOS-HIV 35 scale [37] is an HIV specific quality of life scale and was used to measure mental and physical functioning. Consisting of 35 items, two summary scores can be calculated (physical functioning and mental health) where a high score indicates better health. Alpha scores in this study were both acceptably high: mental functioning α = 0.87 and physical functioning α = 0.92.

#### Depression

Depression was measured with CES-D depression scale [38]. This consists of 20 items scored on a 4-point scale (0–3), 4 of the items are reverse coded. A high score indicates more depressive symptoms. The alpha in this study was 0.93.

#### Posttraumatic Growth

While posttraumatic growth is best measured by identifying the difference in measures over time, this was a cross-sectional study so retrospective growth was measured using Tedeschi & Calhoun’s [31] posttraumatic growth inventory (PTGI) consisting of 21 items. Participants were asked to rate the degree to which they felt they had changed as a direct result of their HIV diagnosis, for example, ‘I have greater appreciation for the value of my own life’. Items are scored on a six- point scale where 0 = no change experienced and 5 = change was experienced to a very great degree. A total ‘posttraumatic growth’ score was calculated where a high score indicates more perceived growth. The alpha for the scale in this study was 0.96.

#### Perceived Stigma

Stigma was measured using Sowell’s stigma scale [39]. This consisted of 13 items measuring internal stigma on a 4-point scale (often, sometimes, rarely and not at all). A total score is calculated and recoding took place so that a high score indicated more perceived stigma. The alpha for this scale was 0.89 in this study.

#### Disclosure Regret

Stigma was also measured with a measure of disclosure regret, consisting of five binominal items which assessed feelings about disclosing, for example ‘After disclosing my HIV status I definitely do not regret it’ (1) or definitely regret it (2). Two items were reverse scored. A total score was achieved by summing all items where a high score indicated more regret. The alpha for this scale in this study was 0.82.

### Analysis

The data were checked for missing data and participants with missing data were excluded from the analysis. Attention checks were made on all scales, two which included reverse-worded items (the CES-D scale and the Disclosure Regret Scale), and there was no evidence of malingering. Hierarchical multiple regression assessed the influence of posttraumatic growth on satisfaction with life, after controlling for perceived stigma and disclosure regret. Block 1 included variables measuring personal characteristics (age, gender, time since diagnosis), functional and mental health, and disease severity and Block 2 included stigma and disclosure regret. Posttraumatic growth was entered in Block 3. Content analysis was used on the open-ended and free response questions.

## Results

### Sample Characteristics

About half the sample identified themselves as gay (45.6%). Slightly more individuals were single or widowed compared to those living with a partner or married. All participants had disclosed their HIV diagnosis to at least one person. Forty-four percent had disclosed to a family member and only 24% had told their current or ex-partner. About 44% had told over 50 people about their HIV status and just over half (56%) had disclosed within a week of their diagnosis (Table 1).

**Table 1:** Demographic characteristics of PLWH

Variable	Number	Percentage
Sexuality		
Heterosexual	32	47.1
Homosexual	31	45.6
Bisexual	5	7.4
Marital status		
Single	34	47.9
Unmarried partner	20	28.2
Married	11	15.5
Widowed	6	8.5
Disclosure of diagnosis	73	100
Relationship to first person disclosed to		
Partner	16	24.2
Family	29	43.9
Friend	18	27.3
Other	3	4.5
Number of people told		
1	2	2.9
< 5	4	5.8
< 10	12	17.4
< 15	7	10.1
< 20	6	8.7
< 30	8	11.6
> 50	30	43.5
Time since disclosure		
1 day	40	57.1
Less than a week	14	20
At least a week	2	2.9
At least a month	2	2.9
At least 6 months	2	2.9
At least a year	10	14.3
Gender		
Male	48	67.6
Female	23	32.4

Note: Missing data accounts for differing totals

Table 2 shows the participants were of working age and were diagnosed with HIV for between one and 30 years. Forty percent scored 16 or lower for depression; the recommended cut off for clinical depression is 16 for this scale, indicating that, with a mean of 20.49, a high proportion (60%) of this sample would be considered clinically depressed. The high means show that posttraumatic change and stigma were perceived by the sample.

**Table 2:** Means and standard deviations of variables

Variable	Range	Means	SD
Age	23–62	40.26	8.14
Time since diagnosis (years)	2–30	11.79	6.83
Life satisfaction	6–35	21.71	7.46
Mental functioning	11–23	18.21	2.90
Physical functioning	20–39	32.3	5.04
Depression	0–48	20.49	13.99
Disease severity	0–73	25.04	17.61
Perceived stigma	13–48	22.98	8.62
Disclosure regret	5–10	6.02	1.54
Posttraumatic growth	0–105	62.83	28.39

Table 3 shows that life satisfaction had positive associations with mental and physical functioning and posttraumatic growth, where those with better functioning and more growth also perceived better life satisfaction. Life satisfaction also showed a negative relationship with depression, disclosure regret and perceived stigma, showing that those with more depressive symptoms, who perceived more regret after disclosing and who perceived more stigma from others, also perceived lower life satisfaction. In addition to this, posttraumatic growth had a significant correlation with disclosure regret, showing that individuals who perceived more regret after disclosing also perceived less posttraumatic growth. In addition, disclosure regret was negatively associated with time since diagnosis and mental functioning, and positively associated with depression and perceived stigma. Perceived stigma was also negatively associated with mental and physical functioning, and associated with more depression, disclosure regret and disease severity. Disclosure regret and perceived stigma are positively and significantly associated showing that the higher the perceived stigma the more the individual regretted disclosing their status to others.

**Table 3:** Bivariate correlations, means and standard deviations

	1	2	3	4	5	6	7	8	9	M	SD
1. Time since diagnosis	–									11.79	6.83
2. Life satisfaction	0.020	–								21.71	7.46
3. Mental functioning	− 0.019	0.656**	–							18.21	2.90
4. Physical functioning	− 0.179	0.403**	0.644**	–						32.3	5.04
5. Depression	0.029	− 0.684**	− 0.820**	− 0.524**	–					20.49	13.99
6. Disease severity	0.045	− 0.306*	− 0.589**	− 0.466**	0.638**	–				25.04	17.61
7. Disclosure regret	− 0.270*	− 0.285*	− 0.317*	0.040	0.342**	0.087	–			6.02	1.54
8. Perceived stigma	− 0.202	− 0.437**	− 0.504**	− 0.301**	0.609**	0.271**	0.423**	–		22.98	8.62
9. Posttraumatic growth	0.054	0.448**	0.252*	0.066	− 0.153	0.170	− 0.458**	− 0.111	–	62.83	28.39

Note: ** < 0.01; * < 0.05

The results in Table 4 show that the most common reason given for disclosing was to receive support. Concern for others’ health was the second most common reason.

**Table 4:** Reasons for disclosure

Reason	Frequency
For themselves	
Needed the support from others	55
To ‘take control’	2
‘Because I am not ashamed’	9
For others	
Concern for others’ health	16
To raise awareness	6

Note: Some people gave more than one answer while others gave no answer

### Predictors of Life Satisfaction

Table 5 below shows that depression and disease severity significantly predicted life satisfaction at Step 1 (all variables at this step accounting for 54.1% of the variance). This shows that more depressive symptoms were associated with less life satisfaction and greater disease severity was associated with more life satisfaction. At Step 2 perceived stigma and disclosure regret were entered into the equation but did not contribute significantly (adding 1% to the variance accounted for). However, once posttraumatic growth was entered into the equation at Step 3 depressive symptoms and posttraumatic growth show a significant relation with life satisfaction, accounting for 6.8% (*p *< 0.001) of the variance. This means that those who perceived more posttraumatic growth and who reported fewer depressive symptoms reported greater life satisfaction. The total model accounted for 62.8% of the variance (*F* (10, 54) = 9.097, *p* < 0.001).

**Table 5:** Regression coefficients for predictors of life satisfaction

Step	ΔR^2^	B	SE	β	95% CI
1.	0.541***				
Time since diagnosis		0.08	0.12	0.07	− 0.15 to 0.31
Age		− 0.07	0.10	− 0.07	− 0.27 to 0.14
Gender		0.77	1.50	0.05	− 2.24 to 3.78
Disease severity		0.11	0.05	0.25*	0.01–0.21
Depressive symptoms		− 0.32	0.09	− 0.59**	− 0.51 to − 1.13
Physical functioning		0.01	0.19	0.01	− 0.36 to 0.39
Mental functioning		0.78	0.46	0.31	− 0.14 to 1.71
2	0.001				
Time since diagnosis		0.08	0.12	0.07	− 0.17 to 0.33
Age		− 0.07	0.11	− 0.08	− 0.28 to 0.14
Gender		0.89	1.60	0.06	− 2.32 to 4.09
Disease severity		0.11	0.05	0.26*	0.01–0.22
Depressive symptoms		− 0.32	0.10	− 0.59**	− 0.52 to − 0.11
Physical functioning		− 0.00	0.20	− 0.00	− 0.40 to 0.39
Mental functioning		0.80	0.48	0.31	− 0.15 to 1.76
Disclosure regret		0.13	0.55	0.03	− 0.97 to 1.23
Perceived stigma		− 0.03	0.11	− 0.03	− 0.24 to 0.19
3	0.086***				
Time since diagnosis		0.11	0.11	0.10	− 0.11 to 0.33
Age		− 0.10	0.10	− 0.11	− 0.29 to 0.10
Gender		0.72	1.46	0.05	− 2.20 to 3.63
Disease severity		0.04	0.05	0.10	− 0.06 to 0.15
Depressive symptoms		− 0.32	0.09	− 0.60***	− 0.51 to − 0.14
Physical functioning		− 0.05	0.18	− 0.04	− 0.41 to 0.31
Mental functioning		0.44	0.45	0.17	− 0.46 to 1.33
Disclosure regret		0.88	0.54	0.19	− 0.21 to 1.97
Perceived stigma		− 0.07	0.10	− 0.08	− 0.27 to 0.13
Posttraumatic growth		0.10	0.03	0.37***	0.04 to 0.15

*p < .05; **p < .01; ***p ≤ .001

## Discussion

As HIV has been reported as a stigmatising illness it seemed likely that disclosure regret may be experienced and stigma might have a negative impact on adjustment. These negative factors may impact negatively on the positive association of posttraumatic growth and adjustment. This paper sought to address this question of whether posttraumatic growth maintains its positive influence on satisfaction with life in PLWH once perceived stigma and disclosure regret were accounted for. Posttraumatic growth showed a positive bivariate correlation with life satisfaction whereas disclosure regret and stigma showed a negative correlation with life satisfaction. These results support other studies showing these relationships [10, 13–15, 27, 28]. Regression analysis showed that, after controlling for stigma and other psychosocial variables, posttraumatic growth was influencing life satisfaction. This showed the positive influence of posttraumatic growth despite the perceived stigma. Perceived stigma and disclosure regret were not influencing life satisfaction. Participants had disclosed to at least one person (usually family or their partner) and most had disclosed close to receiving their diagnosis. This pattern of disclosure is consistent with other research looking at disclosure [40].

That posttraumatic growth was associated with an improvement in life satisfaction shows posttraumatic growth to be important for psychological well-being. This supports the OVT [41] and the literature which shows the positive effects of posttraumatic growth on better psychological health [14, 15, 25, 29, 42, 43]. Knowledge that posttraumatic growth can have a positive impact, despite experiencing stigma and disclosure regret, may be useful for those providing support to PLWH. This knowledge could also be taken into consideration with regard to designing interventions aimed at improving outcomes for this group.

Stigma (including disclosure regret) was not associated with life satisfaction in the final equation and this contradicts other studies looking at stigma in PLWH [3–6]. That stigma did not emerge as a significant predictor is interesting and may relate to the sample as most participants had been diagnosed fairly close to data collection and so may not have been exposed to a lot of stigmatising situations at the time. Disclosure regret has been found in other studies with PLWH, for example, Serovich [44] found nearly 11.6 and 4.3% of their sample experienced regret in disclosing to family and friends respectively, and Nakimuli Mpangula [45] found 23% of their Ugandan sample experienced regret after disclosure to others. This regret has been found to have negative effects on well-being [10, 13–15], however, while we have evidence for the effects of disclosure regret, none of these studies measured posttraumatic growth.

Other predictors of adjustment showed life satisfaction was negatively associated with depression, where those reporting fewer depressive symptoms showed higher life satisfaction scores. This supports previous work looking at depression and wellbeing [46]. These results also support research into health-related quality of life, where psychological well-being and perceived health were associated with life satisfaction in PLWH [47, 48]. The results of this study indicate that improving outcomes for PLWH should focus on support to improve mental health, and to reduce and manage depression. In addition, interventions which make use of posttraumatic growth would help to improve self-report measures of adjustment and well-being.

This study is not without limitations. A longitudinal design would have improved the study allowing for more robust conclusions. The sample was also self-selected and so may have discouraged those who either perceived more stigma or who had not disclosed to anyone from participating. Another limitation was that the perceived stress of the diagnosis was not measured, which may have impacted on the adjustment process and impacted on the results. Future research should also identify different types of disclosure (for example, accidental or intentional).

## Conclusion

To conclude, posttraumatic growth impacted positively on satisfaction with life after controlling for stigma and disclosure regret, showing the strength of this relationship. Stigmatising experiences were also not having a dominating effect in this group. Interventions advising how and when to disclose may help to prevent unnecessary disclosures and feelings of regret, so improving perceptions of health and adjustment. In addition, the positive relationship of posttraumatic growth with life satisfaction would suggest that interventions that include aspects of posttraumatic growth may be beneficial in helping people to adjust to living with HIV.
